# Efficacy of atorvastatin-based treatment in super-aged patients with chronic subdural hematoma: a case series and literature review

**DOI:** 10.3389/fneur.2025.1609514

**Published:** 2025-06-27

**Authors:** Jiangyuan Yuan, Wei Quan, Xuanhui Liu, Pan Li, Jinhao Huang, Chuang Gao, Tao Liu, Yongqiang Zhang, Jianning Zhang, Rongcai Jiang

**Affiliations:** ^1^State Key Laboratory of Experimental Hematology, Laboratory of Post-Neuroinjury Neurorepair and Regeneration in Central Nervous System Tianjin and Ministry of Education, Department of Neurosurgery, Tianjin Neurological Institute, Tianjin Medical University General Hospital, Tianjin, China; ^2^Department of Neurology, Tianjin Huanhu Hospital, Tianjin, China; ^3^Department of Geriatrics, Geriatric Ward of Neurology, Institute of Tianjin Geriatrics, Tianjin Medical University General Hospital, Tianjin, China; ^4^Department of Neurosurgery, Xuanwu Hospital, Capital Medical University, Beijing, China

**Keywords:** chronic subdural hematoma, super-aged patients, atorvastatin, dexamethasone, case series

## Abstract

**Background:**

Chronic subdural hematoma (CSDH) is a common neurological disorder in the elderly, typically managed through surgical intervention; however, in patients aged 90 years and older, surgery is often not feasible due to comorbidities, anticoagulant use, and other age-related factors. This study evaluates the effects of atorvastatin, either as monotherapy or in combination with dexamethasone, in the conservative treatment of CSDH in patients over 90 years old, while also reviewing the current literature on the management of CSDH in this super-aged population.

**Methods:**

Seventeen super-aged patients diagnosed with CSDH at our neurosurgical department between January 2017 and June 2024, who either refused or were considered unsuitable for surgery, were included in the study. Six patients received atorvastatin monotherapy, while 11 were treated with a combination of atorvastatin and dexamethasone. Head imaging scans were analyzed, and the modified Rankin Scale (mRS) and Markwalder’s Grading Scale/Glasgow Coma Scale (MGS-GCS) scores were assessed before and after treatment.

**Results:**

At the six-month follow-up, all patients showed significant improvement in neurological symptoms, as reflected by lower mRS and MGS-GCS scores. Hematomas were completely absorbed in 10 patients, significantly reduced in five, and unchanged in two patients with calcified hematoma. Three patients developed hyperglycemia, and one patient exhibited transaminitis; these adverse effects were resolved following the discontinuation of dexamethasone and the use of hepatoprotective medications. No mortality was recorded during the six-month follow-up.

**Conclusion:**

Our findings suggest that atorvastatin-based treatment may improve the prognosis of CSDH in super-aged patients and offer a viable therapeutic alternative for those ineligible for surgery.

## Introduction

Chronic subdural hematoma (CSDH) is a common neurological disorder in the elderly, typically presenting with headache, hemiparesis, and consciousness disturbances (1, 2). Surgical evacuation is currently the mainstay management for patients with CSDH, including burr-hole drainage, twist-drill craniostomy, and craniotomy (1, 3); however, despite advancements in neurosurgical techniques and operative instruments, management of CSDH in elderly patients remains highly challenging. Advanced age, a primary risk factor for CSDH (4), also correlates with poorer outcomes, likely due to higher comorbidity prevalence and increased use of antithrombotic drugs (5, 6). These factors are associated with higher risks of postoperative recurrence and mortality (5, 7). Moreover, with the population aging, the incidence of CSDH has surged, and the patient demographic is increasingly elderly (8). Therefore, neurosurgeons need to focus more on the treatment of super-aged CSDH patients, especially those over 90 years old.

Surgical intervention has been demonstrated to improve neurological function in CSDH patients over 90 years old who are in good physical condition, but it is associated with a high mortality rate (18.6–26.8% at six-month follow-up) (9, 10). Moreover, many super-aged patients are ineligible for surgery due to complex comorbidities and coagulation disorders, resulting in poor outcomes with conservative care. A study by Hiroyuki Toi, which included 5,414 Japanese CSDH patients over 90 years old, found that more than half had poor neurological outcomes at discharge, and nearly 40% were unable to return home (11). For these patients, particularly those who are ineligible for surgery, there is an urgent need to develop safe and effective conservative treatment strategies.

Several drugs, including dexamethasone, atorvastatin, tranexamic acid, and goreisan, have been investigated for the conservative treatment of CSDH (12). Among these, dexamethasone has been used for over 50 years as either a conservative or postoperative adjunct therapy. However, recent studies suggest that, compared to surgical intervention, dexamethasone treatment results in poorer neurological outcomes and higher complication rates (13), despite its efficacy in preventing postoperative recurrence (14). Nevertheless, it is premature to dismiss dexamethasone’s value in CSDH treatment (15). In contrast, atorvastatin (20 mg daily) has shown significant potential in improving neurological function and promoting hematoma absorption (16), with these effects further enhanced when combined with low-dose dexamethasone (17). As a commonly prescribed lipid-lowering agent, atorvastatin is well-established for preventing cerebrovascular disease in the elderly, with a favorable safety profile.

Therefore, it is reasonable to consider the use of atorvastatin-based conservative treatment for CSDH in super-aged patients, particularly those ineligible for surgery. This case series retrospectively evaluates the clinical outcomes of atorvastatin, either as monotherapy or in combination with dexamethasone, in 17 super-aged CSDH patients who did not undergo surgery. Additionally, we provide a comprehensive review of the existing literature on the management of CSDH in patients aged 90 years and older.

## Methods

### Case series and treatment procedure

This retrospective case study includes consecutive patients aged 90 years or older, diagnosed with CSDH at the Neurosurgery Center of the General Hospital of Tianjin Medical University between January 2017 and June 2024. Patients who underwent surgery or were in palliative care were excluded. A total of 17 super-aged patients who received atorvastatin monotherapy or combination therapy with low-dose dexamethasone were included. Among them, five were deemed ineligible for surgery due to prolonged anticoagulant use, and six were considered unsuitable for surgery based on poor physical condition, as assessed by experienced neurosurgeons. Additionally, two patients experienced postoperative recurrence, and four patients refused surgery due to fear of the procedure (Supplementary Table 1). All patients were fully informed of the alternative therapeutic processes and signed informed consent forms for atorvastatin monotherapy or combined with dexamethasone.

Six patients received atorvastatin monotherapy, including two who were initially treated with a combination of atorvastatin and dexamethasone but were switched to monotherapy due to severe hyperglycemia. The remaining 11 patients underwent a full course of conservative treatment with a combination of atorvastatin and low-dose dexamethasone. For all patients, atorvastatin was administered orally at 20 mg daily, with a minimum treatment duration of 3 months. Additionally, the patients who received combination therapy were orally administered dexamethasone at a gradually decreasing dose (2.25 mg/day for the first 2 weeks, 1.5 mg/day during the third week, and 0.75 mg/day during the fourth week). Atorvastatin therapy was discontinued upon follow-up confirmation of complete neurological symptom resolution.

Follow-up imaging was performed using computed tomography (CT) or magnetic resonance imaging (MRI) at least once after 2 months of treatment. Additionally, a six-month follow-up was conducted through outpatient visits or telephone interviews, during which clinical outcomes were assessed using the modified Rankin Scale (mRS) and the Markwalder Grading Scale/Glasgow Coma Scale (MGS-GCS) (Supplementary Table 2). The MGS is a clinical grading system for CSDH, with scores ranging from 0 (asymptomatic) to 4 (coma with no motor response to painful stimuli) (18). Liver function and blood glucose levels were regularly monitored to promptly detect and manage potential adverse drug reactions.

### Literature review

**Figure 1:**
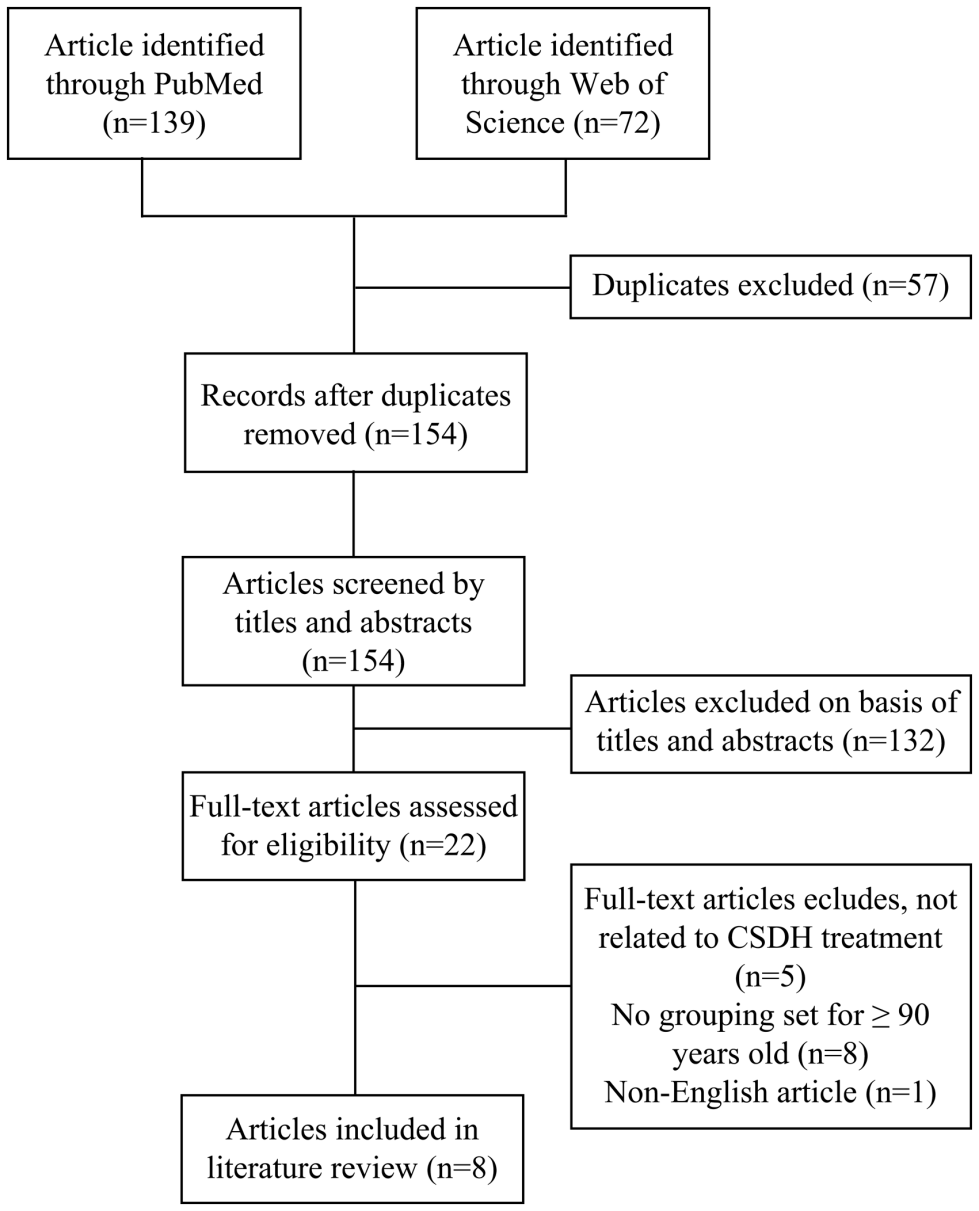
Literature review flowchart. This flowchart delineates the search and review process used to identify and select articles for inclusion in this study.

All relevant articles were identified through PubMed and Web of Science databases using the search terms: “chronic subdural hematoma” or “chronic subdural hemorrhage” and “90 years” or “aged 90” or “nonagenarian.” We reviewed all English-language publications from January 1974 to December 2024. Then, Endnote software was used to remove the duplicate literature between different databases, and a preliminary screening was completed according to the titles and abstracts of the remaining articles. Finally, after excluding literature inconsistent with the review theme through full-text reading, relevant information from eight identified articles was extracted and summarized (Figure 1).

## Results

The mean age of the patient sample was 92.82 ± 3.07 years (range: 90–101 years), and the gender distribution was nearly equal (53% male vs. 47% female). Eight patients (47%) had a history of head trauma prior to the diagnosis of CSDH, four patients (24%) were treated with antiplatelet agents (clopidogrel) for heart disease, and one patient had been on warfarin following cardiac valve replacement before the development of CSDH. Hematomas were predominantly located in the left frontotemporal parietal subdural space, with two patients presenting with bilateral hematomas. Eleven patients (65%) exhibited midline shift due to hematoma compression, including two male patients who experienced postoperative recurrence. Most patients presented with mild to moderate symptoms, including headache, hemiplegia, and mild consciousness disturbances, which allowed for the possibility of conservative treatment. The primary comorbidities included hypertension (11 patients, 65%), diabetes (four patients, 24%), and cardiac disease (six patients, 35%), while a few patients had a history of stroke, deep vein thrombosis, dementia, or malignancy (Table 1).

**Table 1:** Baseline and clinical characteristics of 17 super-aged patients with CSDH.

Case no.	Gender	Age years	Trauma	Recurrent hematoma	Hematoma side	Initial symptoms	Comorbidities	Antithrombotic medicine
1	Female	95	No	No	Right	Headache, Hemiparesis	Hypertension, Diabetes, Previous stroke, Malignancy, Cardiovascular disease	Clopidogrel
2	Female	94	Yes	No	Left	Headache, Cognitive impairment	Hypertension, Cardiovascular disease	-
3	Male	91	No	No	Left	Headache	Diabetes, Cardiovascular disease	Clopidogrel
4	Female	95	No	No	Right	Cognitive impairment, Hemiparesis	Hypertension	-
5	Male	91	No	No	Left	Speech disturbance, Gait disturbance	Hypertension, Diabetes, Cardiovascular disease	-
6	Male	90	Yes	No	Left	Cognitive impairment, Speech disturbance, Gait disturbance	Hypertension, Diabetes, Previous stroke	-
7	Male	98	No	No	Left	Headache	Hypertension	-
8	Male	91	No	No	Left	Gait disturbance	Cardiac valve replacement	Warfarin
9	Male	90	No	No	Left	Hemiparesis	Hypertension	-
10	Female	91	No	No	Left	Speech disturbance, Hemiparesis	Malignancy	-
11	Female	101	Yes	No	Bilateral	Cognitive impairment, Gait disturbance	Hypertension, Dementia, Deep venous thrombosis	Clopidogrel
12	Female	94	Yes	No	Left	Gait disturbance	Cardiovascular disease	Clopidogrel
13	Female	93	Yes	No	Bilateral	Hemiparesis	Hypertension	-
14	Male	92	Yes	No	Left	Hemiparesis	-	-
15	Female	91	Yes	No	Left	Gait disturbance	Hypertension	-
16	Male	90	No	Yes	Left	Cognitive impairment, Hemiparesis, Speech disturbance,	Hypertension	-
17	Male	91	Yes	Yes	Left	Hemiparesis, Speech disturbance	-	-

**Figure 2:**
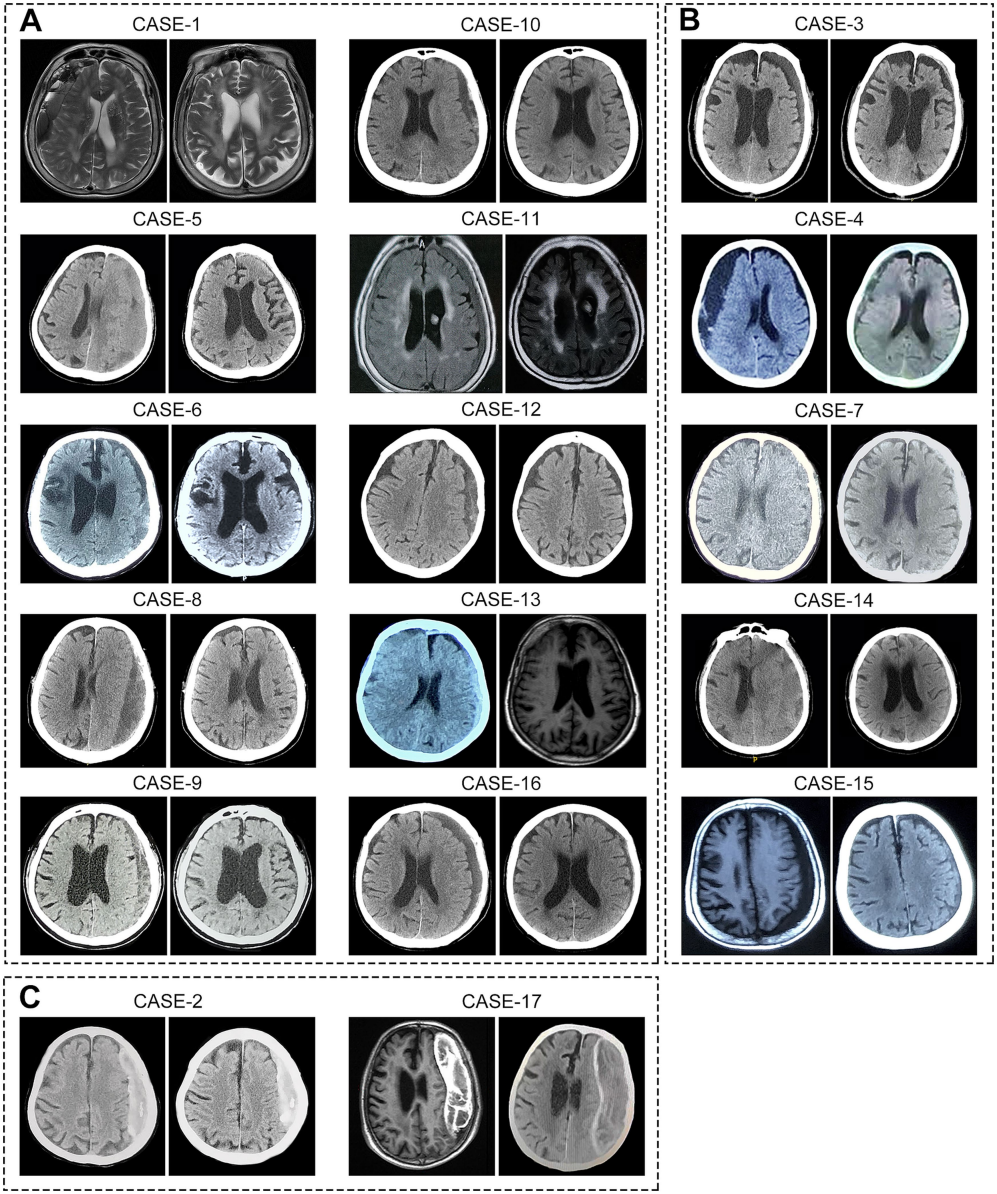
Imaging of hematomas before and after treatment in 17 super-aged CSDH patients. After receiving atorvastatin-based conservative treatment, **(A)** 10 patients showed complete hematoma absorption, **(B)** 5 patients exhibited significant reduction in hematoma size, and **(C)** 2 patients with calcified hematomas showed no apparent absorption.

Following atorvastatin-based conservative treatment, CT/MRI imaging revealed complete hematoma absorption in 10 patients and a significant reduction in five (Figure 2). However, two patients with calcified hematomas showed no evidence of CSDH absorption; despite this, their neurological symptoms fully resolved, and atorvastatin was discontinued at the six-month follow-up. Additionally, compared to initial neurological assessments at the time of CSDH diagnosis, both mRS and MGS-GCS scores showed significant improvement following treatment (Table 2). Upon admission, nine patients (53%) presented with severe disability (mRS score 4–5), all of whom exhibited progressive improvement, with mRS scores reducing to 1–3 after treatment. Likewise, MGS-GCS grades decreased in all 17 patients, with a significant relief of neurological symptoms at the six-month follow-up.

**Table 2:** Outcomes of atorvastatin-based conservative treatment in 17 super-aged patients with CSDH.

Case no.	Treatment method	Hematoma clearance	Midline shift (Y/N)		Modified rankin scale score		Markwalder grading scale /glasgow coma scale score		Adverse drug effects
			Before treatment	After- treatment	Before treatment	After- treatment	Before treatment	After- treatment	
1	ATO	Complete absorption	Y	N	4	2	2	1	N
2	ATO	Calcified	N	N	5	2	3	1	N
3	ATO	Significant absorption	N	N	2	1	1	0	N
4	ATO	Significant absorption	Y	N	4	2	2	1	N
5	ATO	Complete absorption	Y	N	4	1	2	0	Hyperglycemia
6	ATO	Complete absorption	N	N	5	3	3	1	Hyperglycemia
7	ATO + DEX	Significant absorption	Y	N	2	1	1	0	Hepatic injury
8	ATO + DEX	Complete absorption	Y	N	3	0	1	0	N
9	ATO + DEX	Complete absorption	Y	N	3	0	2	0	N
10	ATO + DEX	Complete absorption	N	N	4	1	2	0	N
11	ATO + DEX	Complete absorption	N	N	4	3	3	1	Hyperglycemia
12	ATO + DEX	Complete absorption	N	N	3	0	1	0	N
13	ATO + DEX	Complete absorption	Y	N	3	1	2	0	N
14	ATO + DEX	Significant absorption	Y	N	3	2	2	1	N
15	ATO + DEX	Significant absorption	Y	N	3	1	1	0	N
16	ATO + DEX	Complete absorption	Y	N	4	2	3	1	N
17	ATO + DEX	Calcified	Y	Y	4	3	2	1	N

ATO, atorvastatin; DEX, dexamethasone; Y, yes; N, no.

During atorvastatin therapy, one patient developed elevated transaminase levels, which normalized after treatment with hepatoprotective agents. Additionally, three patients experienced elevated blood glucose levels while receiving concurrent dexamethasone treatment. Of these, two patients with pre-existing diabetes demonstrated poorly controlled glycemia. As a result, dexamethasone was promptly discontinued in these two patients, and atorvastatin monotherapy was continued. Notably, no mortality was recorded during the six-month follow-up period.

A systematic review of the literature identified eight studies on the management of CSDH in patients aged 90 years and older, encompassing 373 patients, 37% of whom were female (Table 3), (9, 10, 19–24). Of these patients, 50–77% had a documented history of head trauma, and 19–64% were on antithrombotic therapy. Despite the lack of established treatment guidelines for this population, the studies suggest that surgical intervention, particularly burr-hole drainage, is safe and recommended for super-aged patients in good physical condition. Compared to conservative care, surgical treatment has been shown to significantly reduce mortality, extend survival, and improve quality of life (9, 19). However, it is associated with higher mortality (8.8–31%) and recurrence rates (10.7–20%) compared to patients under 90 years of age (21–24). Notably, several studies report that regardless of treatment approach, the overall prognosis for super-aged CSDH patients remains poor, with many unable to regain independent living (11, 20). Thus, while surgical intervention is safe and effective for selected super-aged patients in good physical condition, it remains associated with a high mortality rate.

**Table 3:** Current literature on the management of CSDH in super-aged patients aged 90 years and older.

Authors	Patients number	Surgery/ conservative	Surgery outcomes after 6 months			Research conclusions
			Improved	Recurrence	Death	
Stippler et al. (20)	21	16/5	38%	12.5%	31%	Surgical and conservative care both result in poor outcomes.
Tabuchi et al. (19)	20	12/8	N/A	N/A	N/A	Surgery is considered safe and recommended for super-aged patients in good physical condition.
Lee et al. (9)	101	70/31	N/A	12.9%	18.6%	It is beneficial and safe to surgically treat CSDH in super-aged patients.
Bartek et al (23)	75	75/0	N/A	10.7%	13.3%	Surgery result in a higher incidence of 90-day mortality compared to younger patients with CSDH.
Dobran et al (24)	25	25/0	96%	20%	4%	Surgery for CSDH in patients over 90 is as safe and effective as that in patients under 90, but with higher recurrence.
Christopher et al (22)	68	68/0	48.5%	11.8%	8.8%	Surgery result in increased mortality and disability.
Ewbank et al (10)	41	41/0	N/A	N/A	26.8%	Surgery can be safety in selected super-aged patients, but overall mortality is high.
Chiappini et al. (21)	22	22/0	77.3%	18.2%	9.5%	Age was not directly correlated with greater recurrence, postoperative bleeding, or mortality rates.

N/A, not available.

## Discussion

CSDH is a specific type of intracranial hemorrhage, characterized by the accumulation of blood between the dura mater and the arachnoid membrane, resulting in a mass effect that causes headaches, motor deficits, speech difficulties, and cognitive impairments (1). The pathophysiology of CSDH is thought to stem from the rupture of bridging veins following head trauma, leading to hematoma accumulation in the subdural space. This process triggers chronic inflammation, which promotes the formation of hyperpermeable blood vessels and continuous leakage of blood into the subdural space, thereby contributing to CSDH formation (25). This unique pathological mechanism may explain the high recurrence rates of CSDH after surgery and serves as the theoretical basis for using middle meningeal artery embolization (MMAE) in its treatment (26).

Advanced age, anticoagulant use, and severe comorbidities are significant risk factors for recurrent CSDH and are closely associated with poor prognosis (4–7, 27). Notably, complex comorbidities and antithrombotic therapy are prevalent in the super-aged population. In our case series of 17 patients aged 90 years and older, 88% had comorbidities, primarily hypertension, diabetes, and cardiovascular disorders, with 29% receiving antithrombotic therapy. Studies have shown that cardiac and cerebrovascular diseases are the predominant comorbidities in super-aged CSDH patients, with more than half undergoing antithrombotic treatment (10, 23, 24). These factors not only complicate surgical decision-making but also increase patient anxiety regarding surgery, posing significant challenges in the management of CSDH in super-aged patients.

Among the 17 super-aged CSDH patients in our study, 11 were deemed unsuitable for surgery by experienced neurosurgeons due to dependence on antithrombotic therapy or poor physical condition. Additionally, six patients refused surgery, including two who lost confidence following postoperative recurrence and four who were afraid of the procedure. While burr-hole drainage is widely regarded as a safe and effective treatment for CSDH, postoperative recurrence remains a significant challenge in elderly patients. Furthermore, many older patients either cannot tolerate surgery or refuse it, thus being forced into conservative care (28). Compared to surgical intervention, these patients generally have a worse prognosis, with a six-month mortality rate as high as 58.1% (9). Symptomatic supportive therapy alone is insufficient to promote hematoma absorption, and the progression of CSDH further exacerbates neurological deficits, potentially leading to severe complications such as pneumonia, all of which contribute to a worse outcome. However, in this study, 15 patients exhibited significant hematoma absorption and marked improvement in neurological symptoms following atorvastatin-based conservative treatment. A prior randomized controlled trial (RCT) has demonstrated the efficacy of atorvastatin in CSDH management (16). For patients with small hematomas and mild symptoms, atorvastatin-based conservative treatment has gained increasing consensus among neurosurgeons in China (29). This case series is the first to outline the potential effects of atorvastatin monotherapy or its combination with dexamethasone in CSDH patients aged 90 and older.

Dexamethasone has been used as an adjunctive treatment for CSDH for over half a century (30). However, recent studies suggest that, compared to surgery, conservative treatment with dexamethasone is associated with a higher incidence of severe complications, making it less recommended (13). Despite this, dexamethasone has been shown to reduce the recurrence rate of CSDH after surgery significantly (14, 31). This effect likely results from its ability to reduce subdural inflammation, inhibit neovascularization, and decrease blood leakage into the subdural space, thus preventing hematoma reformation (32, 33). Long-term use of high-dose corticosteroids, however, carries risks such as hyperglycemia and immune suppression, which limit their therapeutic effects. Reducing the steroid dosage may provide greater benefits in CSDH treatment. A previous study demonstrated that low-dose dexamethasone (2.25 mg/day, tapered to 0.75 mg/day for 1 month) in combination with atorvastatin yields better results than statin monotherapy in CSDH management (17). Moreover, studies suggested that dexamethasone can promote the absorption of low-density CSDH and improve neurological function (34, 35). Therefore, dismissing the value of corticosteroids in CSDH treatment is premature (15). Given its potential efficacy, further research is needed to assess whether low-dose dexamethasone could be a viable treatment option for super-aged CSDH patients, particularly those ineligible for surgery.

In our study, although two patients experienced postoperative recurrence, their neurological symptoms significantly improved following the initial surgery. In super-aged CSDH patients, burr-hole drainage is currently the primary treatment and markedly improves life expectancy, with six-month survival rates nearly doubling compared to conservative care (9). Despite the clear benefits of surgery, the recurrence rate of 10.7–20% warrants attention (21–24), highlighting the need for optimal perioperative management to prevent recurrence and complications. For patients in good physical condition, local anesthesia may offer better outcomes, as general anesthesia has been associated with a five-fold increase in postoperative complications, primarily pneumonia and cardiovascular events, which are often fatal in elderly patients (36). Furthermore, using body-temperature irrigation fluid during surgery can reduce CSDH recurrence to 6% (37), and 24-h postoperative drainage is more effective in preventing recurrence compared to shorter or longer drainage durations (38, 39). Beyond surgical optimization, postoperative management with atorvastatin or low-dose dexamethasone may further reduce CSDH recurrence (40); however, additional studies are required to establish safe and effective dosing regimens. Given the high prevalence of antithrombotic therapy in super-aged patients, careful consideration is necessary regarding the timing of anticoagulant resumption after surgery. An ongoing pilot RCT investigating the optimal timing for resuming anticoagulation therapy after CSDH surgery may provide valuable insights (41).

Though surgical intervention offers significant benefits, some elderly patients are either ineligible or refuse it. In this cohort of 17 patients receiving conservative treatment, 11 exhibited midline shift due to hematoma compression, yet their neurological deficits were limited to headache, hemiparesis, and mild cognitive dysfunction. Moreover, two patients with calcified hematomas showed substantial neurological improvement at the six-month follow-up, despite no apparent hematoma resolution. These findings suggest that brain atrophy in super-aged patients may alleviate the mass effect, providing a window for conservative treatment. How to promote CSDH absorption? Addressing its pathological mechanisms is crucial. CSDH can be likened to a reservoir, where inflammatory vascular leakage represents the inflow (26), and meningeal lymphatic drainage serves as the outflow (42). Atorvastatin has been shown to preserve endothelial barrier integrity by suppressing inflammation (43, 44). In experimental subdural hematoma models, atorvastatin significantly reduced the expression of pro-inflammatory cytokines such as TNF-*α*, IL-6, and IL-8, and increased the number of anti-inflammatory regulatory T cells within the subdural cavity, thereby attenuating inflammation and vascular permeability (45, 46). These effects were further enhanced by the addition of dexamethasone (17, 47). Moreover, recent studies suggest that meningeal lymphatic drainage is compromised following hematoma formation due to endothelial disruption, whereas atorvastatin treatment can restore lymphatic function and accelerate hematoma clearance (48). Taken together, atorvastatin may facilitate hematoma absorption through a dual mechanism: reducing inflammatory vascular inflow and enhancing meningeal lymphatic outflow. Similarly, MMAE has demonstrated encouraging effects in accelerating CSDH resolution and reducing recurrence by blocking the inflow. (49, 50) Therefore, MMAE may offer a minimally invasive treatment option for super-aged CSDH patients (51).

The aging population has led to a marked increase in the incidence of CSDH, with a notable rise in super-aged patients, posing significant challenges to healthcare systems (8, 52). Future research should focus on gaining a deeper understanding of the pathophysiology of CSDH and developing strategies to prevent its formation and recurrence. Furthermore, there is a critical need to develop safe and effective conservative treatment options for super-aged patients, particularly those ineligible for surgical intervention.

This case study has several limitations. Firstly, it was non-randomized and lacked a direct comparison between conservative and surgical treatment groups. Although the results are promising, it remains unclear whether atorvastatin-based treatment can serve as an effective alternative to surgical intervention in super-aged patients. Additionally, the limited sample size restricts the ability to assess whether a combination of dexamethasone and atorvastatin is more effective than atorvastatin monotherapy. Lastly, this case study included only a six-month follow-up, lacking an evaluation of the long-term effects of atorvastatin-based conservative treatment. Future clinical trials are needed to address these issues and validate the efficacy of conservative treatment strategies for super-aged CSDH patients.

## Conclusion

This retrospective study included 17 super-aged CSDH patients (≥90 years) who were ineligible for surgery. Daily treatment with 20 mg atorvastatin, either alone or in combination with low-dose dexamethasone, led to significant hematoma absorption. Throughout the treatment period, patients underwent close neurological monitoring and cranial imaging. Adverse events included hyperglycemia in three patients and elevated hepatic transaminase levels in one patient, all of which resolved after discontinuation of dexamethasone and initiation of hepatoprotective therapy. At the six-month follow-up, all patients showed notable neurological improvement, with a 100% survival rate. While burr-hole drainage remains the primary treatment for CSDH in super-aged patients, atorvastatin-based conservative therapy may provide a promising alternative for those unable or unwilling to undergo surgery.

## Data Availability

The datasets presented in this article are not readily available because of ethical and privacy restrictions. Requests to access the datasets should be directed to the corresponding authors.
