# Supplementary material for: Efficacy of atorvastatin-based treatment in super-aged patients with chronic subdural hematoma: a case series and literature review
Source: Front Neurol. 2025 Jun 27;16:1609514. doi: 10.3389/fneur.2025.1609514 (PMC12246721; doi:10.3389/fneur.2025.1609514)
Supplement: Supplementary file 1 [file Table_1.pdf]

Supplementary Table 1. Summary of clinical reasons for selecting conservative management in 17 super-aged patients with CSDH.

Case No.	Reasons	
Case 1	Anticoagulant dependence	Unable to underwent surgery due to cardiac insufficiency and anticoagulant dependence.
Case 2	Poor physical condition	Unable to underwent surgery due to cardiac insufficiency.
Case 3	Anticoagulant dependence	Unable to underwent surgery due to cardiac insufficiency and anticoagulant dependence.
Case 4	Poor physical condition	Unable to underwent surgery due to higher frailty.
Case 5	Poor physical condition	Unable to underwent surgery due to cardiac insufficiency and higher frailty.
Case 6	Poor physical condition	Unable to underwent surgery due to higher frailty and severe comorbidities.
Case 7	Surgical phobia	Refusal of surgery for fear of failure and postoperative recurrence.
Case 8	Anticoagulant dependence	Long-term use of warfarin and cardiac insufficiency.
Case 9	Surgical phobia	Refusal of surgery for fear of failure and postoperative recurrence.
Case 10	Poor physical condition	Unable to underwent surgery due to higher frailty and leukemia.
Case 11	Anticoagulant dependence	Unable to underwent surgery due to anticoagulant dependence and higher frailty.
Case 12	Anticoagulant dependence	Unable to underwent surgery due to cardiac insufficiency and anticoagulant dependence.
Case 13	Surgical phobia	Refusal of surgery for fear of failure and postoperative recurrence.
Case 14	Poor physical condition	Unable to underwent surgery due to higher frailty.
Case 15	Surgical phobia	Refusal of surgery for fear of failure and postoperative recurrence.
Case 16	Recurrence	Lost confidence in surgery due to postoperative recurrence.
Case 17	Recurrence	Lost confidence in surgery due to postoperative recurrence.

Supplementary Table 2. Definition of Markwalder's Grading Scale-Glasgow Coma Scale.

Patient's Grade	GCS	Markwalder's Grading Scale
Grade 0	15	Normal neurological status without symptoms.
Grade 1	15	Without neurological deficits, but with symptoms such as headache or unsteady gait.
Grade 2	13-14	Focal neurological deficits, such as drowsiness or disorientation, or variable neurological deficits, such as hemiparesis.
Grade 3	9-12	With stupor but appropriate responses to noxious stimuli and several focal neurological signs such as hemiplegia.
Grade 4	<9	Coma with absent motor responses to noxious stimuli and decerebrate or decorticate posturing.
